# Studies of Reservoir Hosts for Marburg Virus

**DOI:** 10.3201/eid1312.071115

**Published:** 2007-12

**Authors:** Robert Swanepoel, Sheilagh B. Smit, Pierre E. Rollin, Pierre Formenty, Patricia A. Leman, Alan Kemp, Felicity J. Burt, Antoinette A. Grobbelaar, Janice Croft, Daniel G. Bausch, Hervé Zeller, Herwig Leirs, L.E.O. Braack, Modeste L. Libande, Sherif Zaki, Stuart T. Nichol, Thomas G. Ksiazek, Janusz T. Paweska, on behalf of the International Scientific, Technical Committee for Marburg Hemorrhagic Fever Control in the Democratic Republic of the Congo

**Affiliations:** * National Institute for Communicable Diseases, Sandringham, Republic of South Africa; † Centers for Disease Control and Prevention, Atlanta, Georgia, USA; ‡ World Health Organization, Geneva, Switzerland; § University of the Free State, Bloemfontein, South Africa; ¶ Tulane School of Public Health and Tropical Medicine, New Orleans, Louisiana, USA; # Institut Pasteur, Lyon, France; **University of Antwerp, Antwerp, Belgium; †† University of Aarhus, Kongens Lyngby, Denmark; ‡‡ Conservation International, Cape Town, South Africa; §§ Department of Health, Watsa, Democratic Republic of the Congo

**Keywords:** Marburg virus, reservoir hosts, research

## Abstract

To determine reservoir hosts for Marburg virus (MARV), we examined the fauna of a mine in northeastern Democratic Republic of the Congo. The mine was associated with a protracted outbreak of Marburg hemorrhagic fever during 1998–2000. We found MARV nucleic acid in 12 bats, comprising 3.0%–3.6% of 2 species of insectivorous bat and 1 species of fruit bat. We found antibody to the virus in the serum of 9.7% of 1 of the insectivorous species and in 20.5% of the fruit bat species, but attempts to isolate virus were unsuccessful.

## Abstract 2

Marburg virus nucleic acid was found in 12 bats, antibodies were found in 2 species of these bats, but no live virus was isolated.

Marburg virus (MARV) and Ebola virus, members of the family *Filoviridae,* cause outbreaks of severe hemorrhagic fever in Africa. Although humans have on occasion acquired infection from contact with tissues of diseased nonhuman primates and other mammals, the reservoir hosts of the viruses in nature remain unknown.

An outbreak of Marburg hemorrhagic fever ran a protracted course in the gold-mining village of Durba, northeastern Democratic Republic of the Congo, from October 1998 through September 2000. The outbreak involved 154 patients (48 confirmed and 106 suspected cases); the case-fatality ratio was 83% (*1*). Primary cases occurred in young male miners and spread as secondary cases to family members and, less frequently, to healthcare workers and others in the community. Most cases occurred in Durba, but a few secondary cases occurred elsewhere, including nosocomial infections in nearby Watsa village, where severely ill patients sought care. The occurrence of sporadic cases and short chains of human-to-human transmission suggested that infection had been repeatedly introduced into the human population; this suggestion was substantiated by the detection of at least 9 genetically distinct viruses circulating during the outbreak. Identical sequences of MARV were found in patients within but not across clusters of epidemiologically linked cases, although viruses with the same sequences reappeared at irregular intervals during the outbreak. Most (94%) affected miners worked underground in Goroumbwa Mine, rather than in the 7 opencast mines in the village. Cessation of the outbreak coincided with the flooding of Goroumbwa Mine. Interviews with long-term residents and healthcare workers and review of hospital records showed that a *syndrome hémorragique de Durba* [hemorrhagic syndrome of Durba] had been associated with the mine since at least 1987, and a survivor of a 1994 outbreak was found to have antibodies against MARV. The fauna of Goroumbwa Mine included bats, rodents, shrews, frogs, snakes, cockroaches, crickets, spiders, wasps, and moth flies (*1*). We present the results of virus reservoir host studies conducted during the outbreak.

## Methods

In parallel with human epidemiologic studies, visits were made to Durba in May and October 1999 to collect specimens for virus ecostudies. The ecostudies were approved by the International Scientific and Technical Committee for Marburg Hemorrhagic Fever Control, which was coordinated by the World Health Organization on behalf of the government of the Democratic Republic of the Congo. In view of the epidemiologic findings during the outbreak, emphasis was placed on the fauna of Goroumbwa Mine. Bats were caught with mist nets at mine entrances; rodents and shrews were caught live with Sherman traps within and close to the mine; and arthropods (cockroaches, crickets, spiders, wasps, and moth flies, plus streblid, nycteribiid, and mite parasites of bats) were collected by hand or with sweepnets. Vertebrates were euthanized and dissected on site. Blood samples were collected; and samples of liver, lung, spleen, kidney, testes, brain, salivary glands, and fetuses of pregnant females were preserved along with the arthropods in liquid nitrogen dry-shipping containers for transport to the National Institute for Communicable Diseases in South Africa. Extra liver samples were collected for phylogenetic studies on bats and rodents, and formalin-fixed tissue samples were kept for possible histopathologic and immunohistochemical examination. Carcasses were fixed in formalin for α-taxonomy purposes.

Vertebrate tissue and arthropod suspensions were processed and tested for filovirus nucleic acids by reverse transcription–PCR (RT-PCR) and nested PCR by using filovirus-specific large (L) protein gene primers and nested MARV-specific viral protein 35 (VP35) primers as described for samples from human patients during the outbreak (*1*). Nucleotide sequencing of amplicons and sequence data analysis were also performed as described previously (*2*), except that MEGA version 3.1 software was used (*3*). Initial RT-PCR and nested PCR were performed with pooled tissue samples of individual vertebrates; when possible, for specimens that produced positive results, all tissues were retested separately. In attempts to isolate virus as detected by indirect immunofluorescence, suspensions (≈10%) of vertebrate tissues pooled for individual animals and arthropods pooled by species were subjected to 3 serial passages in Vero 76 cell cultures. Serum samples from bats and rodents were tested for antibody to MARV by ELISA by using a modification of the technique described previously for human serum (*1*). ELISA antigen consisted of lysate of Vero cell cultures infected with the Musoke strain of MARV. Bat antibody was detected with antibat immunoglobulin–horseradish peroxidase conjugate (Bethyl, Montgomery, AL, USA) and rodent antibody with antimouse immunoglobulin conjugate (Zymed Laboratories, San Francisco, CA, USA). Net ELISA optical density values were expressed as percent positivity (PP) of a human serum sample confirmed positive for MARV and used as an internal control. Cutoff values for recording positive results were deliberately selected to be stringent at 3 × (mean + 3SD) PP values determined for stored bat (n = 188) and rodent (n = 360) serum samples that had been collected for unrelated purposes in Kruger National Park, South Africa, from 1984 through 1994, and tested at a dilution of 1:100. The Kruger bat samples were collected from 3 species of fruit bats (*Megachiroptera*) and 12 species of insectivorous bats (*Microchiroptera*), including samples from 56 *Chaerephon pumila*, 32 *Rousettus aegyptiacus*, 27 *Mops condylurus*, 16 *Hipposideros caffer,* plus 57 samples from 11 other species.

## Results and Discussion

The numbers of specimens collected, plus the results of RT-PCR, nested PCR, attempts to isolate virus in cell culture, and ELISA antibody determinations, are summarized in the Table. With the exception of a *Nycteris hispida* bat, which was caught near a house in Durba, all specimens were collected within Goroumbwa Mine or its immediate surroundings. An estimated minimum of 10,000 Egyptian fruit bats (*R. aegyptiacus*) roosted in the mine, clustered within the upper galleries. Although the numbers of insectivorous bats were difficult to estimate because these bats roosted mainly in the deeper recesses of the mine, the catch rates indicated substantial numbers of the eloquent horseshoe bat (*Rhinolophus eloquens*) and the greater long-fingered bat (*Miniopterus inflatus*). Few microchiropterans were caught in May, but catch rates improved in October after adjustment of trapping hours and the gauge of mist nets used. Pregnancy was recorded in 12 (24%) of 50 *R. aegyptiacus* females in May and in 2 (4.2%) of 47 females in October; descended testes were found in 2 (6%) of 33 males in May and 19 (25%) of 76 in October. The only indication of breeding activity observed in microchiropterans was that 1/7 *Rh. eloquens* females was pregnant in May.

**Table:** Results from Marburg virus testing of specimens collected in Durba, northeastern Democratic Republic of the Congo, May and October 1999

Species	Total no. sampled	Marburg ELISA antibody, no. positive/no. tested (%)	Filovirus L RT-PCR and virus isolation	Marburg nested VP35 RT-PCR, no. positive/no. tested (%)
Chiroptera: Microchiroptera				
*Hipposideros caffer*	13	0/10	0/13	0/7
*H. commersoni*	17	0/16	0/17	0/13
*Miniopterus inflatus*	38	0/34	0/38	1/33 (3.0)
*Nycteris hispida*	1	0/1	0/1	0/1
*Rhinolophus eloquens*	222	20/206 (9.7)	0/222	7/197 (3.6)
*Rh. landeri*	1		0/1	
Chiroptera: Megachiroptera				
*Lissonycteris angolensis*	3	0/3	0/3	0/3
*Rousettus aegyptiacus*	230	32/156 (20.5)	0/230	4/127 (3.1)
Rodentia				
*Lemniscomys striatus*	10	0/10	0/10	
*Lophuromys sikapusi*	2	0/2	0/2	
*Mastomys natalensis*	4	0/4	0/4	0/1
*Mus (Nannomys) minutoides*	11	0/11	0/11	0/2
*Praomys delectorum*	14	0/14	0/14	0/4
*Taterillus emini*	1	0/1	0/1	0/1
*Rattus norvegicus*	5	0/5	0/5	0/1
Insectivora: Sorcidae (*Crocidura* spp.)	3		0/3	0/3
Amphibia: Anura (unidentified frog)	1		0/1	0/1
Arthropoda: Crustacea (unidentified crab)	4		0/4	0/4
Arthropoda: Hexapoda, Arachnida*	≈2,000		0/22†	

*Cockroaches, crickets, spiders, wasps, moth flies, streblids, nycteribiids, mites. †Pooled specimens.

**Figure 1:**
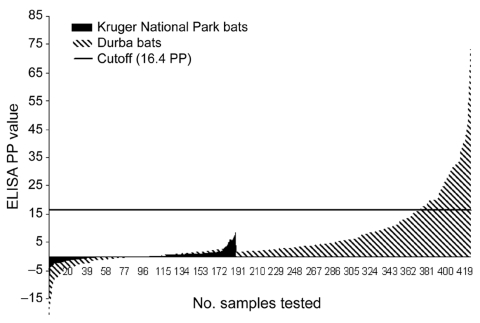
Marburg virus ELISA percent positivity (PP) values recorded on bat serum samples collected in 1999 in Durba, Democratic Republic of the Congo (n = 426), and from 1984 through 1994 in Kruger National Park, South Africa (n = 188). The cutoff PP value of 16.4 was fixed as 3 × (mean + 3 SD) of values observed in the Kruger National Park samples.

The L primer RT-PCR, which was applied to all specimens, produced no positive result. In contrast, the nested MARV VP35 PCR, which was applied only to specimens collected in October 1999, produced positive results on specimens from 12 bats: 1 (3.0%) of 33 *M. inflatus*, 7 (3.6%) of 197 *Rh. eloquens,* and 4 (3.1%) of 127 *R. aegyptiacus*. Nested VP35 PCR on individual tissues of the positive bats produced positive results for liver, spleen, kidney, lung, salivary gland (3/5 bats), and heart (2/5 bats). Attempts to isolate virus in cell cultures from pooled organs were uniformly negative. Applying an ELISA cutoff value of 16.4 PP, determined as 3 × (mean + 3 SD) of values recorded for 188 bat serum samples from Kruger National Park, antibody activity to MARV was detected by ELISA in 20 (9.7%) of 206 *Rh. eloquens* and in 32 (20.5%) of 156 *R. aegyptiacus* serum specimens from Durba (Table; Figure 1). Prevalence of nucleic acid or antibody did not differ significantly between male and female bats or adults and juveniles (determined on the basis of body mass) or between bats collected in May and October. The only RT-PCR–positive bat that had antibody was a *Rh. eloquens* male collected in October. All other investigations produced negative results.

**Figure 2:**
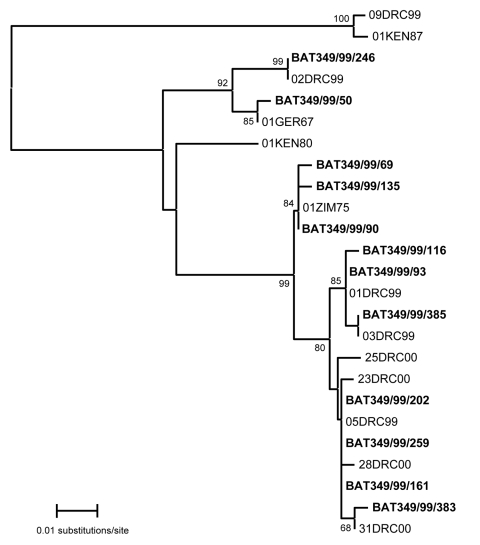
Phylogenetic analysis created by using a neighbor-joining algorithm (MEGA version 3.1, [*3*]) that related sequences of 302-nt fragments of Marburg viral protein 35 gene detected in 12 bats in Durba Mine (**boldface**) to sequences determined for isolates from human patients in the Durba plus previous outbreaks of the disease. Six bat-derived sequences were identical to sequences from human isolates during the outbreak; 1 corresponded to a 1975 human isolate from Zimbabwe, and the remaining 5 represented novel sequences, making a total of 15 distinct MARV sequences found to be in circulation during the Durba epidemic. Bootstrap values were determined by 500 replicates. DRC, Democratic Republic of the Congo; GER, Germany; KEN, Kenya; ZIM, Zimbabwe.

Phylogenetic analysis of the sequences determined for the twelve 302-nt MARV VP35 gene fragments amplified from bat specimens (GenBank accession nos. EU11794–EU118805) showed that 6 corresponded to sequences previously determined for virus isolates from humans during the epidemic (*1*), 1 corresponded to a 1975 human isolate from Zimbabwe, and the remaining 5 represented novel sequences; these last 6 variants from bats, combined with the 9 variants from humans, make a total of 15 distinct MARV sequences found to have been in circulation during the Durba epidemic (Figure 2). Although the differences observed between MARV sequences during the 1999 Durba outbreak were minor, the sequences were consistent in sequential isolates from individual patients and within groups of epidemiologically linked patients (e.g., intrafamilial transmission). In addition, phylogenetic analysis on L gene fragment sequences showed that the 33 virus isolates from patients resolved into exactly the same 9 groups as did the VP35 gene fragments of the same isolates (*1*). Nucleotide sequence divergences of up to 21% observed among the VP35 gene fragments detected in the Durba patients and bats are representative of the diversity of the complete MARV genome and encompass the entire genetic spectrum of isolates obtained over the past 40 years (*1*,*4*). This fact indicates that the virus evolves slowly and that any possible relationship with bats in the Goroumbwa Mine must have extended over a long period. The diversity of MARV sequences detected suggests compartmentalized circulation of virus in bat colonies, as would occur if the species involved existed as metapopulations, spatially discrete subgroups of the same species, as opposed to panmictic populations in which there are no mating restrictions (*5*). Alternatively, bats could be intermediate hosts of the virus.

The history of filovirus outbreaks shows several instances from which it can be inferred that bats may have served as the source of infection. Anecdotal evidence indicates that during shipment from Uganda, the monkeys associated with the first outbreak of Marburg hemorrhagic fever in Europe in 1967 were kept in a holding facility on a Lake Victoria island that had large numbers of fruit bats. In the second filovirus outbreak in 1975, Marburg hemorrhagic fever developed in 2 tourists who had slept in rooms with insectivorous bats at 2 locations in Zimbabwe (*6*). In the first recognized outbreak of Ebola hemorrhagic fever in 1976, the first 6 patients had worked in a cotton factory in Sudan in which insectivorous bats were present (*7*). In 2 separate incidents in 1980 and 1987, infection with MARV was putatively linked with entry into Kitum Cave on the slopes of Mount Elgon in Kenya, where fruit and insectivorous bats are present (*8*,*9*). In 1994, a clan of chimpanzees in a forest reserve in Côte d’Ivoire had been observed feeding in a wild fig tree with fruit bats for 2 weeks before an outbreak of fatal disease, caused by a new strain of Ebola virus, occurred (*10*). The Reston strain of Ebola virus, which is apparently nonpathogenic for humans, was imported into the United States and Europe in infected monkeys from the Philippines; on each occasion, the animals came from a holding facility where they were potentially exposed to the excretions of large numbers of fruit bats (*11*).

The circumstantial evidence in the Marburg hemorrhagic fever outbreak in Durba strongly implicates Goroumbwa Mine as the source of human infection. At least 9 genetic variants of MARV circulated in humans during the outbreak. And because laboratory testing was limited to a few patients, additional variants could have been undetected, as substantiated by our evidence of 6 more variants in bats. The evolution and perpetuation of multiple genetic variants of virus in a fixed location would require a suitably large reservoir host population with constant recruitment through reproduction or migration of susceptible individuals, as generally occurs in small vertebrate and invertebrate populations such as the bat population of Goroumbwa Mine. Failure to isolate live virus may be because it was present in very low concentrations, either early or late in the course of infection. This was the first detection of filovirus nucleic acid and antibody in bats, a phenomenon which was subsequently demonstrated with Ebola virus and MARV nucleic acids and antibodies in fruit bats collected in 2002 and 2005 in Gabon, where it again proved impossible to isolate live virus (*12*,*13*).

The nature of filovirus infection in bats may vary with age and reproductive status. A seasonal pattern in the occurrence of human disease was noted over the 2 years of the epidemic in Durba; transmission began in October–November and peaked in January–February (*1*). In the caves of Mount Elgon in Kenya, Egyptian fruit bats breed in March and September; at other sites in Kenya, the timing varies markedly; no data are available for the Durba area (*14*). The remaining species of bats found in Goroumbwa Mine breed annually, but details for this location are unknown. Thus, although the reproductive status of bats differed in May and October, evidence is insufficient to establish a clear link between breeding patterns of bats in Goroumbwa Mine and the occurrence of Marburg hemorrhagic fever. Nevertheless, many examples in human and veterinary medicine indicate that the outcome of virus infection, development of carrier status, and shedding of virus are influenced by age and reproductive status, including stage of gestation at which infection occurs and the conferral to and duration of maternal immunity in progeny (*15*). Likewise, whether insectivorous bats, fruit bats, or both, are likely to serve as the primary source of infection and whether particular species are involved with secondary transmission of infection to other species is unclear. The evolutionary distinction may exist between cave-roosting bats as hosts of MARV and forest bats as hosts of Ebola virus. Moreover, the ultimate source of infection could prove to be external, such as bat parasites or seasonally active insects in the bats’ diet. Experimental infections in colonized bats could answer some of these questions (*16*).
